# Introducing the “SIMline”—A Simulation Course in the Management of Severe Burns as a Tool in Undergraduate Medical Education

**DOI:** 10.3390/jpm13020338

**Published:** 2023-02-15

**Authors:** Isabel Sawetz, Sophie Hasiba-Pappas, Lars-Peter Kamolz, Judith C. J. Holzer-Geissler, Alexandru Cristian Tuca, David Benjamin Lumenta, Thomas Wegscheider, Hanna Luze, Sebastian P. Nischwitz, Raimund Winter

**Affiliations:** 1 Research Unit for Tissue Regeneration, Repair and Reconstruction, Division of Plastic, Aesthetic and Reconstructive Surgery, Department of Surgery, Medical University of Graz, Auenbruggerplatz 5, A-8036 Graz, Austria; 2 COREMED—Cooperative Centre for Regenerative Medicine, Joanneum Research GmbH, Neue Stiftingtalstr. 2, A-8010 Graz, Austria; 3 Department of Anesthesiology, Medical University of Graz, Auenbruggerplatz 5, A-8036 Graz, Austria

**Keywords:** plastic surgery, reconstructive surgery, burn care, simulation training, medical education, trauma management

## Abstract

*Background:* Management of burn injuries presents a complex and challenging situation for medical staff, especially for inexperienced young doctors. However, training on how to treat burn victims in the clinical setting is rarely taught in undergraduate medical classes. We have created the “SIMline”, a simulation training program explicitly designed for coaching medical students in burn management. *Methods:* A total of 43 students participated in the “SIMline” course, which took place at the training facility at the Medical University of Graz, between 2018 and 2019. The course provided theoretical classes, practical exercises, and a full-scale care process simulation training. The learning progress of the students was monitored via a formative integrated test. *Results:* Students showed great progress throughout the course of the “SIMline” program, as their test scores improved by an average of 88%. The passing rate was 0% at the first exam (prior to course) as compared to 87% at the final exam, taken after the training. *Conclusions:* Comprehensive practical training programs in burn care are underrepresented in medical education. The “SIMline” course presents a novel and effective approach in training medical students in burn management. However, follow-up evaluation is necessary to confirm long-term educational benefits.

## 1. Introduction

Education in burn care in Europe is underrepresented in undergraduate medical curricula. While there is currently no published data on the German-speaking area, is has been reported that only 13% of all medical universities in the UK offer structured education in burn care [1]. In an online questionnaire carried out amongst final year medical students in the UK, over 90% of the 348 respondents stated that they would not feel confident assuming responsibility for initial treatment of a burn victim in an emergency setting [2]. It has also been shown that health care professionals who did participate in any kind of specific burn care training throughout the course of their medical education have a better knowledge of emergency management of severe burns and perform better in simulated burn care exercises in online courses [3]. The burn care trainings mentioned in Breederveld et al. [3] were either the EMSB (Emergency Management of Severe Burns) program, which is specifically designed for burn treatment, or a less burn-focused training as part of the ATLS (Advanced Trauma Life Support). In German-speaking countries, burn care training is currently only offered as part of the ATLS. More targeting programs like the ABLS (Advanced Burn Life Support) or the EMSB originate from the US, Australia or New Zealand, and found their way to Europe through the UK. All the above-mentioned programs have one thing in common: they include simulation courses as part of the training. Simulation training has been proven to be highly effective, not only in management of severe burns but in medical education in general [4]. Nowadays, there is no doubt that simulation-based medical education (SBME) is superior to plain theoretical education in terms of acquisition of clinical skills [5]. Additionally, SBME has shown to increase the effectiveness of daily clinical routine, like surgical ward rounds, enhance management algorithms in burn care and last but not least, improve patient outcome [6]. Simulation training can mimic clinic scenarios in a realistic environment. Even challenging events that may arise in a clinical setting can be trained by improving interdisciplinary management and team building. The goal of simulation training is not to avoid high risk situations, but to acquire the skills to effectively manage these situations when they arise [7].

Simulation training offers a safe and controllable environment in order to train medical personnel in critical and rare situations such as disaster management. One example of crisis management is triage, for instance when operation room (OR) or intensive care unit (ICU) capacities are reached, or when multiple patients in critical condition need to be stabilized at the same time [8]. Over the last years, SBME has become an important element in medical education, especially in residency training [9]. However, only a few examples of comprehensive simulated burn care training courses can be found in the literature [10,11,12,13,14,15].

These mainly focus on coaching medical staff in terms of teamwork and crisis resource management (CRM) [10,11]. To the best of our knowledge, no burn simulation programs specifically directed at undergraduate students have been introduced so far. 

Furthermore, reportedly none of the burn treatment simulation courses implemented a comprehensive simulation pathway that included all necessary steps of patient care—from the arrival at the Emergency Room to discharge from the hospital—which is a called full-scale care process simulation training (FSCPST).

The simulation programs on burn care that can be found in the literature used questionnaires that had been filled out by the participants as an objective assessment. Moreover, they primarily focused on the evaluation and quality of the course, rather than the medical knowledge acquired by it [10,11,16].

## 2. Methods and Development

### 2.1. Educational Objective

The “SIMline”, which was first introduced as an elective subject in the undergraduate medical curriculum of the Medical University of Graz, is a simulation course on how to treat severe burn victims. The primary objective is to impart basic knowledge in burn care to undergraduate medical students, focusing on both surgical and anesthesiologic aspects, as well as to encourage them to further incorporate that knowledge by offering a FSCPST. During the simulation process students are educated in CRM by practicing communication skills and teamwork. The secondary objective during the “SIMline” course is to train the students in specific clinical, medical and surgical skills as an element of the part-task training. As an example, participants can practice surgical interventions (like escharotomies) on mannequins serving as simulated burn victims, as well as surgical airway installation, bronchoscopy, chest tube insertion or fiberoptic endotracheal intubation.

### 2.2. Facilities

The “SIMline” course takes place at a medical training and simulation center (Clinical Skill Center, “CSC”) at the extent of 814 m^2^ in Graz, Austria. The training center is equipped with 2 trauma rooms, 1 neonatal/pediatric trauma room, 2 procedure rooms, 1 operating room (with an adjacent scrub room), 2 ward rooms and 1 intensive care unit. The CSC provides all necessary equipment for a realistic simulated training in immediate clinical trauma management (intubation material, surgical instruments, surgical scrubs and gowns, defibrillators and many more). This training center is also equipped with mannequins, on which the students can practice. There are different mannequins for every clinical scenario—such as pediatric live support, cardiovascular examination or full-scale trauma management for ATLS simulation. 

### 2.3. Course Design

The “SIMline” course is specifically designed for students from the Medical University of Graz. A total of 30 students can participate in the training program (30 students per round). For the practical part of the course, students are divided into small groups of 4 to 6 participants. The course is organized, held and supervised by plastic surgeons and anesthesiologists (attendings as well as residents) from the Medical University of Graz. 

As an algorithmic scenario training, the “SIMline” program is inspired by courses that have already been established, like the ATLS and the ESBM. The “SIMline” program, however, combines both theory and practice over a period of 5 days, for a total of 21 h. In contrast to previously described burn care specific simulation courses [10,11,12,13,14,15], the “hands on” portion entails a full-scale care process simulation training (FSCPST). FSCPSTs focus on the entire process of patient care, starting with the initial assessment in the emergency room (ER) and proceeding with diagnostic, therapeutic and logistic steps and decisions—such as following patient transfer to the OR or ICU. It continues with the act of critical care transfer, surgical treatment in the OR and/or intensive care until and concludes with the discharge of the patient. Furthermore, FSCPSTs consist of high-fidelity training modalities [17].

The 3 big components of the course are:

1. a theoretical session for 2 days of 4,5 h each day (9 h in total)
2. a practical part for 2 days of 4 h each (8 h in total)
3. a full-scale care process simulation training (FSCPST) for 1 day (4 h in total)

### 2.4. Theory

The first 2 days of the course consist of theoretical lectures, covering basic knowledge of emergency treatment of severe burns concerning both surgical and anesthesiologic aspects. The main topics are: Burn injury care in the preclinical setting, at the ER, the OR, the ICU and the ward, critical care transfer, airway management, analgosedation and intraosseous vascular access. Each topic is covered in a lecture of 45 min, followed by a 15-min break. 

### 2.5. Practice

#### 2.5.1. Part-Task Training

Part-task training (PTT) is an important tool to teach specific medical or surgical skills, which are part of complex algorithms, by breaking them down into divided tasks. Students can hereby get familiar with medical tools as well as surgical instruments and procedures [17]. As examples for PTT skills acquired in the “SIMline” course, students are taught proper airway management such as bag-mask-ventilation, endotracheal intubation or even cricothyrotomy. As for surgical expertise, students learn how to perform an escharotomy on mannequins or practice tangential excision of predefined areas in foam wound suture pads (Limbs&Things.com, Savannah, GA, USA). Furthermore, they can even practice to harvest split thickness skin grafts (STSGs) with dermatome simulators on a human skin imitate made out of MicrofoamTM tape (3M Health Care) [18] and porcine skin [19].

#### 2.5.2. Algorithm Training

Algorithm training (AT) helps to give structure to complex situations in medicine. One example for successful AT is the Advanced Trauma Life Support (ATLS) course [20]. The complex treatment of a trauma patient broken down into its individual tasks. Execution and order of these consecutive treatment steps are structured according to a distinct algorithm. In AT, participants perform a series of tasks following a certain structured guideline, part of which is improving communication skills and teamwork. These “soft skills” are particularly important for emergency treatment in burn care, especially for interdisciplinary decision-making and managing unexpected events or disruptions [17]. In the “SIMline” course, algorithm trainings for tasks like “Intraosseous Vascular Access” and “Analgosedation” are offered. Every AT commences with an introductive briefing and concludes with a debriefing, which allows students to reflect on their achievements, their mistakes, and possibilities of improvement. 

**Figure 1:**
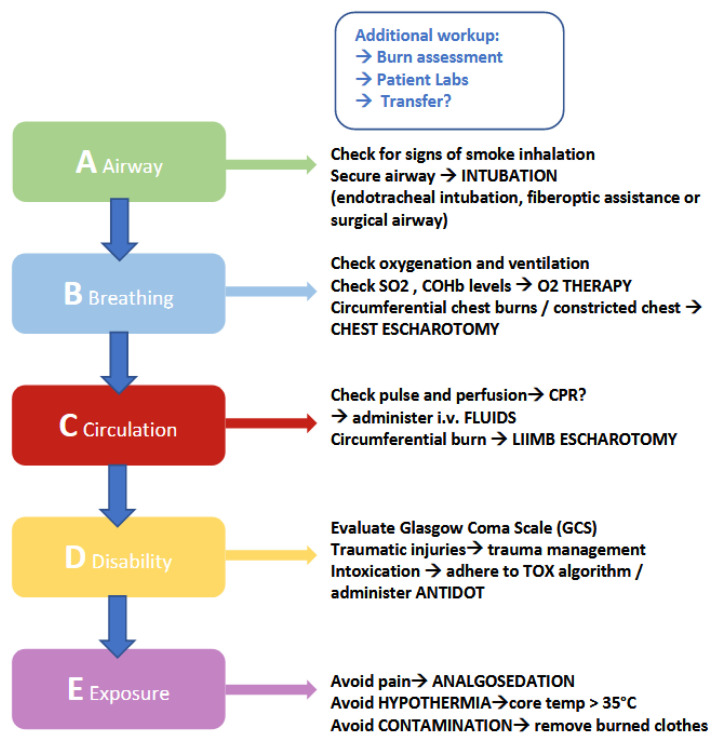
Burn algorithm: Simplified schematic display of important steps in burn management. This table raises no claim to completeness. Although this is an original graphic created for this publication, similarities to existing figures may apply.

The algorithm training for burn care, which is taught and applied throughout this course, follows the basic concept of the “ABCDE” model (see Figure 1). The “ABCDE” model, which is an acronym for assessing the patient according to Airway-Breathing-Circulation-Disability-Environment, is a great example for algorithmic medical practice. The purpose of this “step-by-step” protocol is to facilitate and optimize a patient’s initial examination, especially in a situation that requires immediate medical attention, by assessing the patient in a structured and comprehensive manner. At the Medical University of Graz, trauma and intensive care management is taught according to the ATLS system, which includes the “ABCDE” algorithm. The “SIMline” training for immediate burn management implements and combines the algorithmic approach of ATLS with current guidelines for burn management with the aim to provide a well-rounded education in burn care for medical students. Examples for burn-specific steps in AT are early intubation, chest and/or limb escharotomy, fluid administration according to specific formulas and adequate analgosedation to decrease the patients’ pain and consciousness to a therapeutically beneficial degree. The substances used in this situation are predominantly as Fentanyl, Midazolam and/or Ketamine. 

#### 2.5.3. Scenario Training

The scenario training (ST) combines part task training (PTT) and algorithm training (AT) with a background story, invented to create a more realistic scenario. This type of training aims to encourage students to apply the knowledge previously acquired through PTT and AT by implementing it in the management of different simulated “real-life” situations, whilst simultaneously mastering purposely incorporated obstacles and distractions. In the “SIMline” ST, medical students get the opportunity to demonstrate what they have learned from theoretical lessons and practice their technical and communication skills taught during PTT and AT.

#### 2.5.4. FSCPST

Full-scale care process simulation training consists of high-fidelity training modalities and concentrates on the entire process of the patient care [17]. In the “SIMline” course, this means that the students must take the lead on patient management, starting with the initial assessment of the trauma patient in the emergency room (ER), and continuing with interdisciplinary decisions, such as transportation of the patient to the operation room (OR) or the intensive care unit (ICU). Surgical skills such as performing an escharotomy and/or excising predefined burned areas are practiced in the OR, intensive care management such as airway problems and fluid loss is handled at the ICU. The FSCPT includes all steps of burn treatment protocols, the final stage being the postoperative care in the patient ward until patients can be discharged. 

The students are being observed by the lecturers during the simulation training. The supervisors’ role is to monitor, not to intervene, since the goal of the training is to let students apply the knowledge they had previously gained by theoretical lessons in the simulation on their own—without assistance from the lecturers. 

The FSCPST in the “SIMline” program was documented by a videographer. The edited video demonstrating the brief process of FSCPST can be accessed via the Supplementary Materials (Video S1). 

### 2.6. Objective Evaluation

At the very beginning of the course, before theoretical lessons start, students must take a formative integrated test (FIT) consisting of 26 questions concerning basic medical knowledge about emergency treatment of severe burns. Both surgical and anesthesiologic aspects are included. The test is no multiple-choice test, as it is designed in a “question—answer” format, which means the students must write down their answers. A maximum score of 64 points can be achieved if all questions are answered correctly.

One week after the simulation course is completed, students take the formative integrated test (FIT) for a second time. They are handed out the exact same test as before, the questions are identical. It is important to mention that students are not informed about the quiz beforehand, hence they have no time to specifically prepare for it. To objectively evaluate the learning progress of the students throughout the “SIMline” program, their test results (grades) from the pre- and post-training exams are compared.

## 3. Results

The “SIMline” simulation training has been carried out twice, once between January (theoretical part) and February (practical part) 2018 and once between January and February 2019. A total of 43 undergraduate medical students participated, 24 of which completed the course in 2018, 19 of which in 2019.

**Figure 2:**
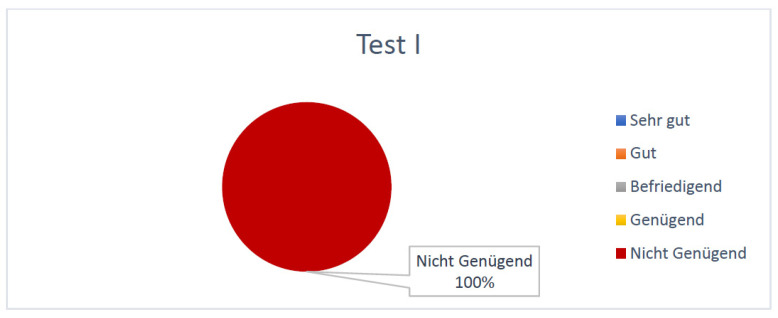
Percentual grade distribution of tests taken by the students before the SIMline course.

At the first test (taken before the simulation training) students achieved an average of 23.4 points (±6.1) out of a maximum score of 64. According to the equivalence key for grades (Table 1) all the students (100%) received the lowest grade, “Nicht genügend”/“F” (Figure 2).

**Figure 3:**
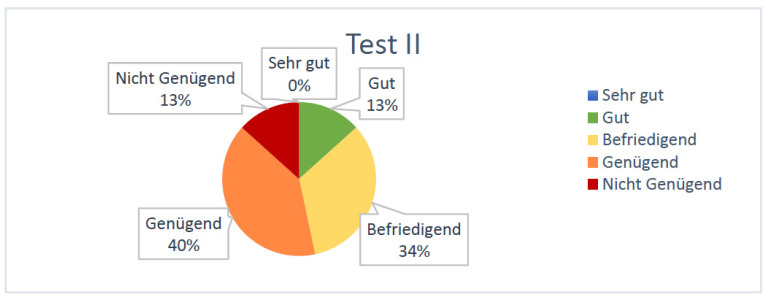
Percentual grade distribution of tests taken by the students after the SIMline course.

At the second test (taken after the simulation training) students achieved an average of 44.1 points (±6.1) out of 64 and thus showed an average improvement of 88%. According to the equivalence key for grades, only 13% of the students were assessed with “Nicht genügend”/“F” after the second exam (Figure 3). The same percentage of participants was graded with a “Gut”/“B” after the final test, over 1/3 received a “Befriedigend”/“C” and 40% improved to a “Genügend”/“D”.

## 4. Discussion

The acute management of a severely burned patient presents a complex and stressful clinical situation for medical staff. Unfortunately, specific training in burn injury management is underrepresented in undergraduate medical curricula [1,2]. The outcome of a burn victim not only determined by medical knowledge and competence, but is also influenced by factors like interdisciplinary communication, which affects teamwork and decision-making. These skills are important, especially in a stressful situation like trauma care where taking immediate action is required. As we have gathered from the literature, the know-how necessary to master trauma management cannot be learned from books or theoretical lectures alone. Practical experience—such as simulation training—is imperative in the educational process [5]. Evidence shows that many adverse events in medicine result from miscommunication and ineffective teamwork rather than from lack of medical knowledge [21]. This underlines the importance of implementing the principles of Crisis Resource Management (CRM) as a fundamental element of medical education, even in undergraduate curricula, so that students can acquire skills like decision-making, interdisciplinary teamwork, communication and leadership in addition to theoretical medical expertise early on. Growing evidence shows that traditional, exclusively clinical education is insufficient if the goal is skill acquisition and optimizing patient safety [5]. Effective training in CRM can be helpful in increasing patient safety by preventing errors in patientcare [22,23,24].

The “SIMline” course combines both theoretical lessons with a high-fidelity simulation training over a total training period of 5 days. To our knowledge based on the currently available literature, this is the first burn care simulation course that is directed at specifically at undergraduate medical students and that contains a FSCPT—meaning the burn victim is treated from the point of admission at the ER until the final discharge from hospital, including all treatment steps (OR, ICU, postoperative treatment at the ward).

The objective assessment of students’ improvement was performed with the help of a formative integrated test (FIT). In the first exam, which students completed before the first lesson of the program, all participants (100%) received the lowest grade, “Mangelhaft”, which is equivalent to an “F” in US grading. It should be noted that the grading system for the FIT might have been too demanding and may need to be revised in the future, since none of the students managed to achieve the best grade “Sehr gut”/“A” even at the end of the course. Given that students showed an average improvement of 88%, which was statistically significant, in respect to their FIT grades after participating in the “SIMline” program, we feel confident in stating that the course was successful in broadening their knowledge on burn care protocol. Furthermore, students were asked to share their personal experience throughout this course in order to gain a more nuanced impression of the efficacy of the program and identify potential flaws and opportunities for improvement. The response we received from the participants was extremely positive. Students especially profited from the FSCPST part of the “SIMline” program. This feedback is consistent with the evaluation and subjective impression of the supervisors. They described a high level of participation and enthusiasm and observed great progress in the students in terms of knowledge, skill and teamwork throughout the course.

Although the objective assessment and personal evaluation were very promising, there are some limitations to this project. One of the most important ones might be the lack of follow-up. There were no additional exams, questionnaires or training sessions after the course was concluded, therefore no statement can be made about long-term learning success and effectiveness of the training program. A reevaluation of the students’ level of theoretical and practical knowledge would give more insight on the sustainability of the program. Another aspect that could be improved in the future is the evaluation method during the simulation course. Although the students were observed by the supervisors and given verbal feedback after the simulation, the objective assessment of the learning process was predominantly measured with the help of the written exam.

When comparing our methods to other simulated burn management programs, we noticed that some authors, like Gasteratos et al. [25] and Lam et al. [26], applied a comprehensive evaluation checklist in order to assess the participants’ practical performance. Each practical skill, such as endotracheal intubation or peripheral venous access, was evaluated and graded individually. Implementing this evaluation method in the “SIMline” program would allow a more objective and in-depth assessment of the students´ practical progress in the future. Furthermore, it might be helpful in identifying the participants´ “weak spots”, so that the supervisors can focus on the tasks that appear especially challenging or require more training.

A review of current literature revealed very few studies that are similar to our project in terms of design and execution. Although simulation-based burn care training was reported a few times, the study design showed high variety. Baird et al. [27], for example, relied on tablet-based simulation training, whereas Pywell et al. [28] used actors, that had been painted by professional make-up artists to resemble real-life burn victims, in their course. Zhang et al. [29] combined video-based learning with a hands-on setting, however this course focused solely on the indications and steps of escharotomies. Highly realistic simulation training on mannequins, as demonstrated in the “SIMline” program, was applied by Reeves et al. [30] and D´Asta et al. [10].

Overall, authors reported positive feedback from the participants and objectively improved performance after simulation training. A major disparity between most of these training programs and the “SIMline” course is that they primarily targeted medical personnel, such as surgical residents or nurses, that already has experience in the field of trauma care. These findings coincide with the statement that simulation-based burn care training is still underrepresented in undergraduate medical education.

The majority of medical students has no experience in this area. Most of them have never even practiced clinical tasks, such as tracheotomies, escharotomies, intraosseous vascular access or “minor” plastic surgeries such as local flaps, before. In our “SIMline” course, undergraduate students get the opportunity to learn and improve these skills in a safe environment that allows room for questions and mistakes. In order to ensure patients’ safety, it is sometimes preferable to let students learn new procedures on mannequins before entering the workplace and performing them on real patients. An emergency scenario such as handling a critical burn patient in the ER can be quite overwhelming for medical students and inexperienced doctors, but the real-life clinical setting does not provide the time for practice or the room for mistakes that a simulation training has to offer. The FSCPST implemented in the “SIMline” course allows students to experience and master such stressful (simulated) events, which helps prepare them for real-life emergencies that may occur throughout the course of their medical career.

As McGaghie et al. [5] stated in their meta-analysis on simulation based medical education: “The purpose of medical education at all levels is to prepare physicians with the knowledge, skills, and features of professionalism needed to deliver quality patient care.”

We strongly believe that the “SIMline” course fulfils this exact purpose in the field currently underrepresented burn care training in undergraduate medical studies.

## 5. Conclusions

Educational training programs concerning the treatment of burn victims are currently underrepresented in Europe, especially in the curricula of medical universities. In this study, we introduce the “SIMline” course, one of the first burn care simulation training programs, created specifically for emergency treatment of burn care victims and directed at undergraduate medical students. This course combines both theoretical and practical education, which includes a full-scale care process simulation training (FSCPST). The “SIMline” course has successfully been conducted twice (in 2018 and 2019). The effectiveness and learning curve of the participants were measured by a written exam specifically created for this program. Results showed that students performed significantly better after completing the course, as their average test scores improved by 88%. By further promoting the “SIMline” course as part of the medical curriculum for undergraduate medical students, we aim to improve and broaden undergraduate medical education in burn care.

## Figures and Tables

**Table 1:** Equivalence key for grades according to achieved points in formative integrative test (FIT) ^1^.

Grade	Percentage	Points	Equivalence to US-Grades
Sehr gut (1)	100–91%	65–59	A
Gut (2)	90–83%	58–54	B
Befriedigend (3)	82–74%	53–48	C
Genügend (4)	73–66%	47–43	D
Nicht genügend (5)	<66%	<42	F

^1^ Equivalence key for grades according to achieved points in formative integrative test (FIT). In Austria, grades reach from “1”/“A” to “5”/“F” in descending order, with “1” being the best/highest and “5” being the worst/lowest grade. Receiving a “5” is equivalent to failing the exam.

## Data Availability

Availability of supplementary files is presented under “supplementary materials”. All sources used for this article are stated under “references”. Any other data may be requested from the authors directly.

## Supplementary Materials

The following supporting information can be downloaded at: https://www.mdpi.com/article/10.3390/jpm13020338/s1, We submitted a short video in order to demonstrate the process of the full-scale care process simulation training (Video S1: Simulation training). It is available at: None of the persons portrayed in this Video can be identified, sequences where the faces were visible were edited (faces blurred beyond recognition).
